# Advanced Esophageal Squamous Cell Carcinoma in Young Female Patient With Durable Complete Response on Treatment

**DOI:** 10.7759/cureus.15255

**Published:** 2021-05-26

**Authors:** Natalia Jankarashvili, Tamar Melkadze, Mariam Tchiabrishvili, Armaz Mariamidze, Giorgi Arveladze

**Affiliations:** 1 Department of Radiation Oncology, Academician Fridon Todua Medical Center, Tbilisi, GEO; 2 Department of Clinical Oncology, Academician Fridon Todua Medical Center, Tbilisi, GEO; 3 Department of Pathology, Pathology Research Center, Tbilisi, GEO; 4 Department of Radiation Oncology, David Tvildiani Medical University, Tbilisi, GEO

**Keywords:** esophageal squamous cell carcinoma, female, chemoradiotherapy, locally advanced, gender role

## Abstract

Esophageal carcinoma is the seventh most common cancer and the sixth most lethal cancer worldwide. There are two main histological types of esophageal carcinoma: adenocarcinoma (AC) and squamous cell carcinoma (SCC). Both histological types are more common in males than females. Menopause is an independent risk factor for esophageal cancer while usage of hormonal therapy (estrogen plus progesterone) is associated with a lower risk of esophageal SCC in postmenopausal women. Gender differences have an impact on SCC incidence, however, it is unclear if gender has a prognostic value for survival. The present case report describes a young woman who developed SCC of the esophagus. The disease was diagnosed in the locally advanced stage. Definitive chemo-radiotherapy induced complete response. These findings might suggest that in young women esophageal SCC may have a better prognosis.

## Introduction

Esophageal carcinoma is the seventh most common cancer and the sixth most lethal cancer worldwide. A high incidence rate is evident in Eastern Asia. Incidence rates of esophageal cancer are 9.3 per 100,000 in men and 3.5 per 100,000 in women worldwide [1]. Risk factors for esophageal cancer include smoking, Barrett’s esophagus, alcohol consumption, hot tea drinking, human papilloma virus (HPV) and poor oral health, low intake of fresh fruit and vegetables, and low socioeconomic status [2]. There are two main histological types of esophageal carcinoma: adenocarcinoma (AC) and squamous cell carcinoma (SCC). Both histological types are more common in males than females. Menopause is an independent risk factor for esophageal cancer, while the usage of hormonal therapy (estrogen plus progesterone) is associated with a lower risk of esophageal SCC in postmenopausal women. Breast-feeding history also plays a protective role against esophageal cancer, particularly among white women [3,4]. Gender difference has an impact on SCC incidence, however, it is unclear if gender has a prognostic significance for the outcome. We herewith report the case of a 38-year-old female patient with a history of esophageal SCC.

## Case presentation

A 38-year-old female presented with a one-year history of progressive dysphagia and fatigue. The patient had no history of smoking or secondhand smoking exposure, alcohol consumption, and tobacco chewing. She was reproductive, with two pregnancies and two childbirths. She did not have a family history of cancer or chronic diseases. The patient was exposed to massive amounts of wood smoke seven years ago. She experienced a loss of appetite for the last several months and confirmed a weight loss of 17% of her body weight in the last two months. The patient had dysphagia for solids and liquids. On physical examination, no abnormalities were found and her body mass index (BMI) was 18.3.

**Figure 1:**
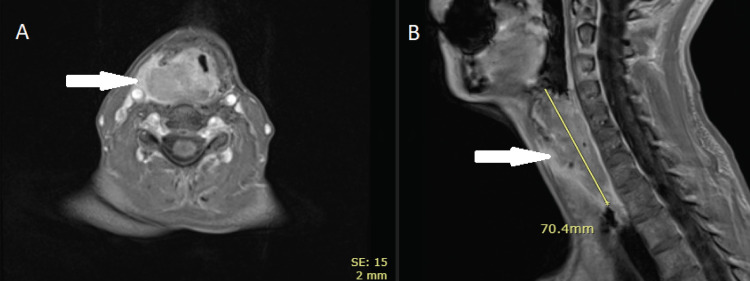
Pre-treatment magnetic resonance imaging performed before starting the chemoradiation Axial (A) and sagittal (B) T1-weighted MRI show tumors growing from the esophagus, invading the larynx.

The patient then underwent clinical and radiological examination. Neck and chest computed tomography (CT) with intravenous contrast demonstrated esophageal tumor mass, longitudinally measuring 50 mm with no nodal or metastatic disease. Cervical magnetic resonance imaging (MRI) revealed a 70 mm tumor spreading from the hyoid bone to the esophagus, invading the larynx. No lymphadenopathy was detected (Figure 1).

**Figure 2:**
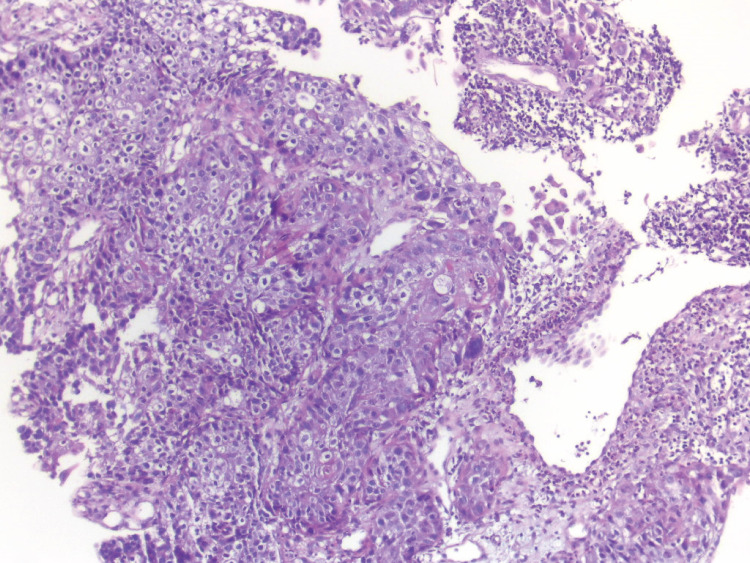
Endoscopic biopsy section, X200, hematoxylin and eosin staining The proliferation of atypical squamous cells marked nuclear pleomorphism and a lot of mitotic figures.

Abdominal and pelvic CT scans and routine laboratory tests were without pathological findings. Esophagogastroduodenoscopy (EGD) showed a lesion with the size of about 70 mm, starting from 17 cm from incisors on the left wall of the cervical esophagus with an exophytic growth pattern and deformation of the left vocal cord. Biopsy revealed poorly differentiated esophageal SCC (Figure 2). The tumor was staged clinically as T4N0M0.

The patient underwent chemotherapy (CHT) using Paclitaxel 75 mg/m^2^ (mg per square meter of body-surface area) and carboplatin AUC2 (area under the curve) weekly for a total of six weeks with concurrent radiotherapy (RT) for a total dose of 66 Gy, in 33 fractions, daily dose of 2.0 Gy/fraction. At the beginning of the treatment, the patient could only swallow liquids with small amounts, which requested per os hyperalimentation with fluids (oral solution, Nutridrink protein, daily dose of 125 ml) that provide high-value protein and energy. Additionally, the patient got intravenous fluids with volume expanders-normal saline and lactated Ringer's solution.

**Figure 3:**
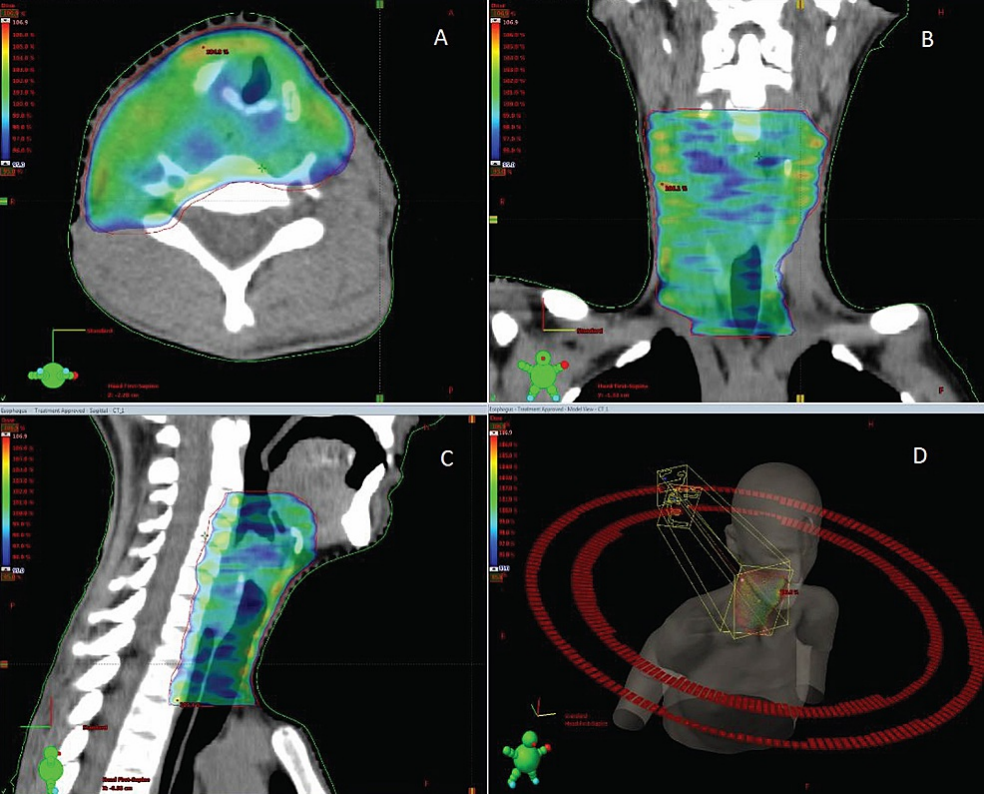
Radiation therapy treatment volumes Axial (A), coronal (B), and sagittal (C) view of dose distribution; (D) 3D view of the target volume. CTV includes gross tumor, cervical part of the esophagus, and periesophageal region. PTV includes CTV+0.5 cm. PTV coverage is adequate. CTV: clinical target volume, PTV: planning target volume, GTV: gross tumor volume.

After CT simulation, target volume and organs at risk (OAR) were delineated. The gross tumor volume (GTV) was determined by a combination of findings on endoscopy, CT, and MRI. Clinical target volume (CTV) included GTV, cervical part of the esophagus, and periesophageal region. The planning target volume (PTV) was generated with a 5 mm symmetrical margin around the CTV (Figure 3). Lungs, tongue, heart, spinal cord, normal esophagus were defined as OAR. The patient was treated on the True Beam Linac with 6 MV photons and the set-up was daily checked by using a cone-beam CT image. Her symptoms relieved after seven fractions of RT. By the end of the treatment course, she was able to swallow even solid food.

The patient tolerated the treatment well. The treatment course was notable for esophagitis, with moderate pain (Common Terminology Criteria for Adverse Events, CTCAE, v4.0: G2), nausea (CTCAE v4.0: G1), and vomiting (CTCAE v4.0: G1), which was successfully managed conservatively.

**Figure 4:**
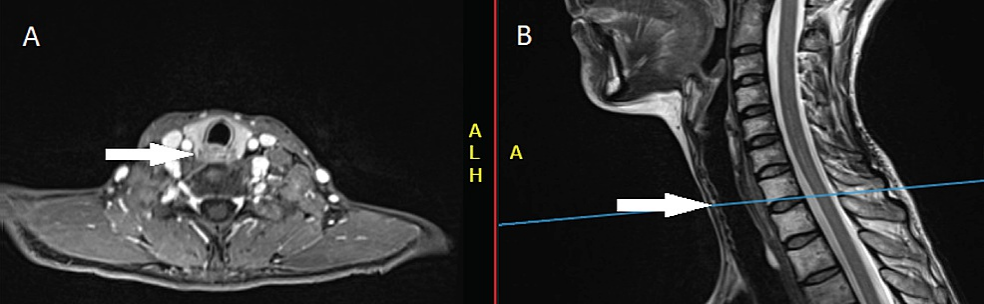
Post-treatment Imaging performed six weeks after completing the chemoradiation Axial (A) and sagittal (B) T1-weighted MRI shows a complete response to the treatment.

Cervical MRI, chest CT and EGD were performed six weeks after completion of definitive chemo-RT. Investigations showed a complete response to the treatment. Imaging revealed postradiation fibrotic changes (Figure 4) and biopsy confirmed the absence of malignant cells.

**Figure 5:**
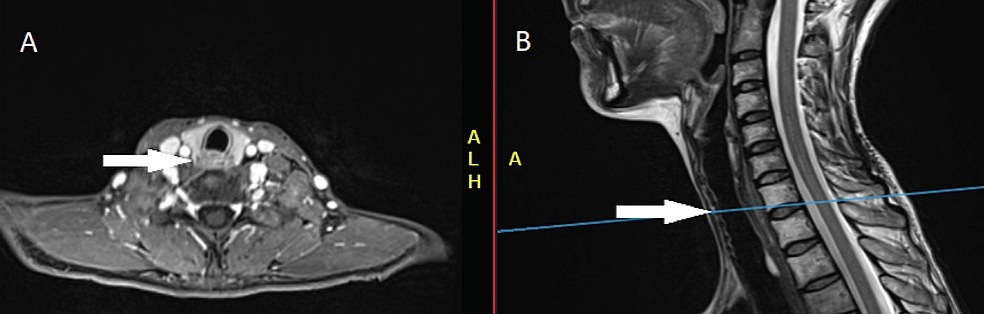
Follow-up imaging performed 24 months after completing the chemoradiation Axial (A) and sagittal (B) T1-weighted MRI still show a complete response to the treatment.

The patient continued surveillance with CT or MRI imaging and EGD. After 24 months of close follow-up, the patient remains cancer-free (Figure 5).

## Discussion

The present case report may indicate that in young women esophageal SCC might have a good prognosis even with locally advanced disease. Donohoe et al. have shown that patients younger than 50 years had better rates of disease-specific survival than older patients; however, younger patients were more likely to be treated curatively rather than palliatively. However, in one study, inferior survival was associated with patients aged less than 35 years [5]. van Nistelrooij et al. presented no significant difference in the five-year survival between patients <50 and >50 years of age (P>0.05). In this study, younger patients, however, presented with more advanced disease stages and received more frequent treatments, which means that younger patients had surgery more often as compared to older patients: 40.6% versus 37.9%, P=0.047 [6].

Our case raises an important question of whether the female gender confers a favorable prognosis in patients with esophageal cancer. Recently, it was demonstrated that estrogen stimulates apoptosis in esophageal squamous cancer cells. In the case of esophageal AC, malignant cells respond to treatment with selective estrogen receptor ligands that result in decreased cell growth and apoptosis [7]. Furthermore, esophageal SCC is an estrogen-dependent human malignancy. Estrogen receptor β (ERβ) immunoreactivity in malignant cells carries an unfavorable prognosis for SCC patients so that ERβ might become a target molecule for esophageal SCC therapy [8] and a recent Swedish study seems to have confirmed that women with esophageal SCC had a better prognosis independent of treatment [9].

One of the possible risk factors for the development of SCC could have been fire-wood smoke exposure. Wood smoke contains high levels of polycyclic aromatic hydrocarbons (PAH), which may lead to gastrointestinal carcinogenesis [10]. According to Kayamba et al., exposure to biomass smoke is associated with the development of upper gastrointestinal cancers [11]. Furthermore, wood burning has been linked to esophageal SCC, and it was observed that wood smoke was more strongly associated with SCC among women than men [12].

Chemo-RT effect in our patient was dramatic. We hypothesize SCC radiosensitivity would be favorably affected by gender. According to previous studies, androgen exposure can promote the growth of malignant cells of SCC. Androgen receptors can lead to the progression of esophageal SCC [13,14]. Luo et al. also demonstrated that sex was an independent prognostic factor in patients with esophageal SCC who underwent definitive radiotherapy, with better survival outcomes for women than men [15]. Further studies are needed to show if hormonal status determines sensitivity to RT. As estrogen and progesterone levels are high in young female patients, it may provide a good prognosis in this subgroup of patients.

## Conclusions

In conclusion, our patient with esophageal SCC had a long-term complete response after definitive chemo-RT. Our case highlights the possible importance of independent risk factors: female gender and young age in the favorable prognosis of esophageal SCC. Since estrogen decreases tumor cell growth and leads to apoptosis and androgen promotes tumor progression, young female patients may have much more benefit from the treatment because of high estrogen and low androgen levels. We also suggest that SCC radiosensitivity may also be favorably affected by gender. However, there are not enough data to emphasize the current issue, so further studies are needed.

## References

[1] (2021). GLOBOCAN 2018: oesophagus cancer fact sheet. http://gco.iarc.fr/today/data/factsheets/cancers/6-Oesophagus-fact-sheet.pdf.

[2] Mao WM, Zheng WH, Ling ZQ (2011). Epidemiologic risk factors for esophageal cancer development. Asian Pac J Cancer Prev.

[3] Bodelon C, Anderson GL, Rossing MA, Chlebowski RT, Ochs-Balcom HM, Vaughan TL (2011). Hormonal factors and risks of esophageal squamous cell carcinoma and adenocarcinoma in postmenopausal women. Cancer Prev Res (Phila).

[4] Wang BJ, Zhang B, Yan SS (2016). Hormonal and reproductive factors and risk of esophageal cancer in women: a meta-analysis. Dis Esophagus.

[5] Donohoe CL, MacGillycuddy E, Reynolds JV (2011). The impact of young age on outcomes in esophageal and junctional cancer. Dis Esophagus.

[6] van Nistelrooij AM, van Steenbergen LN, Spaander MC, Tilanus HW, van Lanschot JJ, Lemmens VE, Wijnhoven BP (2014). Treatment and outcome of young patients with esophageal cancer in the Netherlands. J Surg Oncol.

[7] Sukocheva OA, Wee C, Ansar A, Hussey DJ, Watson DI (2013). Effect of estrogen on growth and apoptosis in esophageal adenocarcinoma cells. Dis Esophagus.

[8] Zuguchi M, Miki Y, Onodera Y (2012). Estrogen receptor α and β in esophageal squamous cell carcinoma. Cancer Sci.

[9] Kauppila JH, Mattsson F, Brusselaers N, Lagergren J (2018). Prognosis of oesophageal adenocarcinoma and squamous cell carcinoma following surgery and no surgery in a nationwide Swedish cohort study. BMJ Open.

[10] Abedi-Ardekani B, Kamangar F, Hewitt SM (2010). Polycyclic aromatic hydrocarbon exposure in oesophageal tissue and risk of oesophageal squamous cell carcinoma in north-eastern Iran. Gut.

[11] Kayamba V, Heimburger DC, Morgan DR, Atadzhanov M, Kelly P (2017). Exposure to biomass smoke as a risk factor for oesophageal and gastric cancer in low-income populations: A systematic review. Malawi Med J.

[12] Mlombe YB, Rosenberg NE, Wolf LL (2015). Environmental risk factors for oesophageal cancer in Malawi: A case-control study. Malawi Med J.

[13] Matsuoka H, Sugimachi K, Ueo H, Kuwano H, Nakano S, Nakayama M (1987). Sex hormone response of a newly established squamous cell line derived from clinical esophageal carcinoma. Cancer Res.

[14] Dong H, Xu J, Li W (2017). Reciprocal androgen receptor/interleukin-6 crosstalk drives oesophageal carcinoma progression and contributes to patient prognosis. J Pathol.

[15] Luo HS, Xu HY, Du ZS, Li XY, Wu SX, Huang HC, Lin LX (2019). Impact of sex on the prognosis of patients with esophageal squamous cell cancer underwent definitive radiotherapy: a propensity score-matched analysis. Radiat Oncol.

